# Association between teeth loss and nasogastric tube feeding dependency in older adults from Taiwan: a retrospective cohort study

**DOI:** 10.1186/s12877-021-02596-x

**Published:** 2021-11-12

**Authors:** Chun-Chieh Wang, Yu-Yen Chen, Kuo-Chuan Hung, Shang-Jung Wu, Yung-Feng Yen, Chu-Chieh Chen, Yun-Ju Lai

**Affiliations:** 1 Division of Chest Medicine, Department of Internal Medicine, Puli Branch of Taichung Veterans General Hospital, Nantou, Taiwan; 2 Department of Eldercare, Central Taiwan University of Science and Technology, Taichung, Taiwan; 3 School of Medicine, National Yang-Ming University, Taipei, Taiwan; 4 Department of Ophthalmology, Taichung Veterans General Hospital, Taichung, Taiwan; 5 School of Medicine, Chung Shan Medical University, Taichung, 402 Taiwan; 6 Community Medicine Research Center and Institute of Public Health, National Yang-Ming University, Taipei, Taiwan; 7 National Chung Hsing University, Taichung, Taiwan; 8 Department of Anesthesiology, Chi Mei Medical Center, Tainan, Taiwan; 9 Department of Nursing, Puli Branch of Taichung Veterans General Hospital, Nantou, Taiwan; 10 College of Nursing Taichung, Central Taiwan University of Science and Technology, Taichung, Taiwan; 11 Department of Health Care Management, National Taipei University of Nursing and Health Sciences, No.365, Ming-te Road, Peitou District, Taipei City, 112 Taiwan; 12 Section of Infectious Diseases, Taipei City Hospital, Taipei City Government, Taipei, Taiwan; 13 Institute of Hospital and Health Care Administration, National Yang-Ming University, Taipei, Taiwan; 14 Department of Exercise Health Science, National Taiwan University of Sport, Taichung, Taiwan; 15 Division of Endocrinology and Metabolism, Department of Internal Medicine, Puli Branch of Taichung Veterans General Hospital, No.1, Rongguang Rd, Puli Township, Nantou County, 545 Taiwan

**Keywords:** Teeth loss, Nasogastric tube feeding, Older people

## Abstract

**Background:**

To examine the association between teeth loss and nasogastric tube feeding dependency in older people.

**Methods:**

The National Health Interview Survey (NHIS) 2005, 2009, and 2013 in Taiwan. Participants were selected by a multistage stratified sampling method and baseline characteristics, including socioeconomic status and health habits, were obtained by well-trained interviewers. The NHIS was linked with the National Health Insurance research database 2000–2016 and the National Deaths Dataset, which contains all the medical information of ambulatory and inpatient care. Cox regression was used to examine the association between the number of teeth lost and nasogastric tube feeding dependency.

**Results:**

There were 6165 adults older than 65 years old enrolled in the analysis, with 2959 male (48%) and the mean (SD) age was 73.95(6.46) years old. The mean follow-up duration was 6.5(3.3) years. Regarding the teeth loss categories, 1660 (26.93%), 2123 (34.44%), and 2382 (38.64%) of participants were categorized as having no teeth loss, loss of 1–9 teeth, and loss of 10–28 teeth, respectively. During 39,962 person-years of follow-up, new-onset nasogastric feeding dependency was recognized in 220(13.25%), 256(12.06%), and 461(19.35%) participants who were categorized as having no teeth loss, loss of 1–9 teeth, and loss of 10–28 teeth, respectively. Kaplan-Meier curves demonstrated significant findings (Log-rank *P* < 0.01). After potential confounders were adjusted, compared with those without teeth loss, older adults who had lost 10–28 teeth had significantly increased risks of occurrence nasogastric feeding dependency (AHR, 1.31; 95% CI, 1.05–1.62; *p*-value = 0.02). Furthermore, a significant dose-response relation between the number of teeth lost and increased risk of nasogastric feeding was found (p for trend< 0.01).

**Conclusions:**

Older adults who had lost 10–28 teeth had a significantly increased risk of nasogastric tube feeding dependency. Early identification of the oral disease is crucial for the prevention of the occurrence of teeth loss and the following nutrition problems, which would reduce risk of nasogastric tube feeding dependency.

## Key points

1. Older adults who had lost 10–28 teeth had a significantly increased risk of nasogastric tube feeding dependency.
2. Older age, male gender, consuming vegetables and fruits less than 5–7 days/week, heart failure, stroke, and cancer were also significant risk factors of nasogastric tube feeding dependency.
3. It is important to maintain good oral health to prevent loss of teeth and further occurrence of nasogastric tube feeding dependency.

## Background

In Taiwan’s aging society, dysphagia is a common problem among older adults such as older nursing home residents [1], older adults admitted to the hospital [2], patients with stroke [3], and even older adults who live independently [4]. People with dysphagia and poor nutrition status usually need to supplement with enteral nutrition. Enteral feeding can be carried out by nasogastric, nasal duodenal, and nasojejunal routes. In Taiwan, the most commonly used enteral feeding is nasogastric tube feeding.

According to the report from the Ministry of Health and Welfare Taiwan, the prevalence of nasogastric tube feeding among people older than 65 years is 17.9% in 2020 (19.8% in men and 16.2% in women) [5]. This rate is only 11.6% in Japan and 6.6% in Germany. In America, the proportion of nasogastric tube feeding in nursing home residents with advanced dementia declined from 11.7% in 2000 to 5.7% in 2014 [6, 7]. In Taiwan, around 99% residents were covered under the National Health Insurance program [8]. The low cost and convenience for the caregiver to feed the patients cause the high prevalence of nasogastric tube feeding in Taiwan.

However, it was reported that enteral feeding did not prevent aspiration pneumonia, improved bed sore healing, or reduced infection rates, or prolong survival [9]. The presence of nasogastric, nasal duodenal and nasojejunal feeding tubes can also promote negative psychosocial characteristics, such as depression and loss of social contact associated with tube feeding [10].

Any difficulty in the swallowing pathway can be defined as dysphagia. People with anatomical or physical defects in the oral cavity, pharynx, larynx, and esophagus may experience dysphagia. The anatomy and physiology of swallowing changes with increasing age. Loss of muscle mass power and connective tissue elasticity leads to loss of strength and variety of motion [11].

Teeth loss can cause changes in oral anatomy. Previous research reports that dysphagia and swallowing difficulty due to aging are more prevalent in older adults with more teeth loss [12]. However, the association of teeth loss and nasogastric tube feeding dependency is seldom discussed. We thus aimed to discover the association of teeth loss and nasogastric tube feeding dependency by using data from the National Health Interview Survey (NHIS), the National Health Insurance research database and the National Death dataset.

## Methods

### Data collection

The Health Promotion Administration, Ministry of Health and Welfare in Taiwan performed a national health survey since 2001. The National Health Interview Survey (NHIS) chose participants by the multistage stratified systematic sampling method. Baseline information of socioeconomic status and health habits were collected by well-trained interviewers. The NHIS was conducted every 4 years since 2001. We used data from the NHIS in 2005, 2009, and 2013. The NHIS was linked with the National Health Insurance research database 2000–2016 and the National Deaths Dataset. The National Health Insurance research database contains all the medical information, including ambulatory and inpatient medical records. This research was approved by the Research Ethics Committee. All methods were performed in accordance with the relevant guidelines and regulations.

### Teeth loss

The number of teeth lost was collected by asking the question “Except wisdom teeth, is there any one tooth been lost or extracted?” and the response item was: 0. No; 1. Yes, how many teeth?___; 2. Complete denture.

### Nasogastric tube feeding dependency

Nasogastric tube feeding dependency was confirmed by the procedure code “47017C” and “47018C” from the National Health Insurance research database 2000–2016. Participants were defined as having nasogastric feeding dependency if the procedure code was coded three or more times in the outpatient or inpatient medical records consecutively in six months.

### Potential confounders

Baseline characteristics of the participants, including socioeconomic status and health habits, were collected by well-trained interviewers. The classification of body mass index was recommended by the Health Promotion Administration of the Ministry of Health and Welfare. Comorbidities were recognized from the National Health Insurance research database 2000–2016. Disease diagnosis was coded with the International Classification of Disease, 9th Revision Clinical Modification (ICD-9-CM). Comorbidity was defined if the diagnosis code was noted in three or more outpatient medical records or one outpatient medical record before the occurrence of nasogastric tube insertion. Comorbidities were documented, including chronic renal failure (ICD-9-CM code 585), heart failure (ICD-9-CM codes 428), liver cirrhosis (ICD-9-CM codes 571.2, 571.5), stroke (ICD-9-CM code 430–438), and cancer (ICD-9-CM code 140–208).

### Study design

This was a nationwide retrospective cohort study. We selected older adults aged more than 65 years old from the NHIS 2005, 2009, and 2013. Those compatible with the definition of nasogastric feeding dependency from the NHIRD before the date of interview were excluded. The follow-up period was calculated since the interview date and censored on the date of nasogastric feeding dependency, death, or Dec 31, 2016.

### Statistical analysis

First, survival curves were illustrated. The Kaplan–Meier method with log-rank test was applied to compare the differences across the three categories of teeth loss. Participants with no teeth loss were used as the reference group. Hazard ratios (HRs) and 95% confidence intervals (CIs) for nasogastric feeding dependency were calculated by Cox proportional-hazards regression models. Data analyses were performed by using SAS 9.4 (SAS Institute, Cary NC).

## Results

**Fig. 1:**
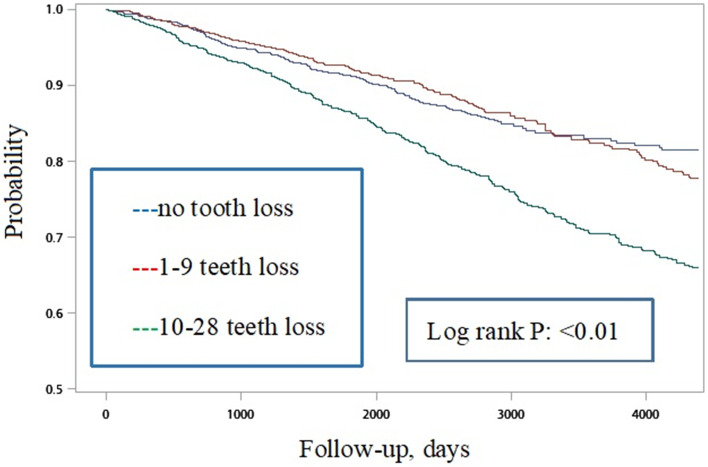
Kaplan–Meier survival curve estimates for nasogastric feeding dependency among three groups of teeth loss for the older population in Taiwan

There were 7382 participants older than 65 years who took part in the three rounds of the NHIS in 2005, 2009, and 2013. After excluding those with unavailable information on teeth loss (*n* = 972) and those already diagnosed with nasogastric feeding (*n* = 245), 6165 participants were included in the analysis. Among them, 2959 were male (48%) and the mean (SD) age was 73.95(6.46) years old. The mean follow-up duration was 6.5(3.3) years. Figure 1 illustrates the Kaplan–Meier curves of the three categories of teeth loss, which revealed a significant difference (*p*-value of log-rank test < 0.001).

Table 1 summarizes the baseline characteristics and comorbidities of study participants and the result of the univariate Cox regression model. Regarding the teeth loss categories, 1660 (26.93%), 2123 (34.44%), and 2382 (38.64%) of participants were categorized as having no teeth loss, loss of 1–9 teeth, and loss of 10–28 teeth, respectively. During 39,962 person-years of follow-up, new-onset nasogastric feeding dependency was recognized in 220(13.25%), 256(12.06%), and 461(19.35%) participants who were categorized as having no teeth loss, loss of 1–9 teeth, and loss of 10–28 teeth, respectively. Compared with those with no teeth loss, participants with more teeth loss had increased risks of nasogastric feeding dependency (loss of 1–9 teeth: hazard ratio [HR], 1.03; 95% confidence interval [CI], 0.86–1.24; *p*-value =0.74; loss of 10–28 teeth: HR, 1.73; 95% CI, 1.46–2.07; p-value< 0.001). Older age, male gender, underweight, widowed/divorced/separated, low education level, current and former smokers, lower fruit and vegetable intake, betel nut chewing habit, and those with comorbidities were correlated with a significant risk of nasogastric feeding dependency.

**Table 1:** Baseline characteristics and results of univariate Cox regression analysis (*n* = 6165; 937 nasogastric feeding dependency cases)

Demographics	Total (% in column)	Number of nasogastric feeding dependency cases (% in row)	Hazard Ratio	(95% CI)	***p***-value
Number of teeth lost					
0	1660 (26.93%)	220(13.25%)	Ref		
1–9	2123 (34.44%)	256 (12.06%)	1.03	0.86–1.24	0.74
10–28	2382 (38.64%)	461 (19.35%)	1.73	1.47–2.03	< 0.001
Age in years, mean (SD)	73.95(6.46)	77.26(6.88)	1.10	1.09–1.11	< 0.001
Gender					
Female	3206 (52%)	439 (13.69%)	Ref		
Male	2959 (48%)	498 (16.83%)	1.28	1.12–1.45	< 0.001
Body mass index (kg/m^2^)					
Underweight (< 18.5)	248 (4.38%)	55 (22.18%)	1.69	1.28–2.24	< 0.001
Normal weight (18.5–23.9)	2679 (47.29%)	415 (15.49%)	Ref		
Overweight (24–26.9)	1623 (28.65%)	204 (12.57%)	0.77	0.65–0.91	0.003
Obesity (≥ 27)	1115 (19.68%)	168 (15.07%)	0.95	0.80–1.14	0.61
Marriage status					
Married/cohabiting	4011 (65.09%)	541 (13.49%)	Ref		
Never married	123 (2.00%)	26 (21.14%)	1.70	1.15–2.52	0.01
Widowed/divorced/separated	2028 (32.91%)	370 (18.24%)	1.47	1.29–1.68	< 0.001
Education					
Low (elementary or below)	4146 (72.88%)	707 (17.05%)	Ref		
Moderate (junior/senior high)	1068 (18.77%)	131 (12.27%)	0.77	0.64–0.92	0.01
High (college or above)	475 (8.35%)	55 (11.58%)	0.70	0.54–0.93	0.01
Household income					
<US$952/month	2848 (58.15%)	452 (15.87%)	Ref		
US$952–2222/month	1439 (29.38%)	226 (15.71%)	1.00	0.85–1.17	1.00
>US$2222/month	611 (12.47%)	83 (13.58%)	0.88	0.70–1.11	0.28
Smoking status					
Never	4382 (71.09%)	613 (13.99%)	Ref		
Current	839 (13.61%)	150 (17.88%)	1.27	1.06–1.52	0.01
Former	943 (15.30%)	173 (18.35%)	1.48	1.25–1.75	< 0.001
Alcohol consumption					
No	4170 (77.99%)	675 (16.19%)	Ref		
Less than once a week	740 (13.84%)	79 (10.68%)	0.65	0.52–0.83	< 0.001
More than once a week	437 (8.17%)	68 (15.56%)	0.80	0.62–1.02	0.07
Vegetable					
< 5 days/week	422 (6.86%)	133(31.52%)	Ref		
5–7 days/week	5730 (93.14%)	793(13.83%)	0.41	0.34–0.50	< 0.001
Fruit					
< 5 days/week	1401 (22.77%)	339 (24.20%)	Ref		
5–7 days/week	4751 (77.23%)	587 (12.36%)	0.61	0.53–0.70	< 0.001
Betel nut					
Never	5552 (90.07%)	844 (15.20%)	Ref		
Current	223 (3.62%)	28 (12.56%)	0.81	0.56–1.20	0.30
Former	389 (6.31%)	64 (16.45%)	1.20	0.93–1.55	0.16
Chronic renal failure					
No	5889 (95.52%)	865 (14.69%)	Ref		
Yes	276 (4.48%)	72 (26.09%)	2.54	2.00–3.23	< 0.001
Heart failure					
No	5600 (90.84%)	788 (14.07%)	Ref		
Yes	565 (9.16%)	149 (26.37%)	2.35	1.97–2.80	< 0.001
Liver cirrhosis					
No	6052 (98.17%)	911 (15.05%)	Ref		
Yes	113 (1.83%)	26 (23.01%)	1.93	1.31–2.86	< 0.001
Stroke					
No	4798 (77.83%)	621 (12.94%)	Ref		
Yes	1367 (22.17%)	316 (23.12%)	2.13	1.86–2.44	< 0.001
Cancer					
No	5549 (90.01%)	822 (14.81%)	Ref		
Yes	616 (9.99%)	115 (18.67%)	1.47	1.21–1.79	< 0.001

*Abbreviations*: *SD* standard deviation, *CI* confidence interval

Table 2 summarizes the result of the multivariate Cox regression model. Risk factors associated with the occurrence of nasogastric feeding dependency were identified. After controlling for potential confounders, compared with those with no teeth loss, older people who had lost 10–28 teeth had significantly increased risks of incident nasogastric feeding dependency (AHR, 1.31; 95% CI, 1.05–1.62; *p*-value = 0.02). A significant dose-response association between the number of teeth lost and increased risk of nasogastric feeding was found using the trend test (p for trend< 0.01). Other variables that increased the risk of incident nasogastric feeding dependency included age, male gender, consuming vegetables and fruits less than 5–7 days/week, heart failure, stroke, and cancer. The number of teeth lost was used as a continuous variable and the result of multivariable Cox regression showed that older adults with who had lost more teeth had a significantly increased risk of nasogastric feeding (AHR, 1.01; 95% CI, 1.003–1.017 *p*-value = 0.008).

**Table 2:** Results of multivariate Cox proportional hazards analysis of incidence of nasogastric feeding dependency

Demographics	Adjusted Hazard Ratio	(95% CI)	***p***-value
Number of teeth lost^a^			
0	Ref		
1–9	1.02	0.81–1.29	0.87
10–28	1.31	1.05–1.62	0.02
Age (years)	1.09	1.07–1.10	< 0.001
Gender			
Female	Ref		
Male	1.31	1.06–1.63	0.01
Body mass index (kg/ m^2^)			
Underweight (< 18.5)	1.11	0.78–1.58	0.56
Normal weight (18.5–23.9)	Ref		
Overweight (24–26.9)	0.95	0.78–1.15	0.58
Obesity (≥ 27)	1.02	0.82–1.27	0.87
Marriage status			
Married/cohabiting	Ref		
Never married	1.47	0.94–2.32	0.09
Widowed/divorced/separated	1.06	0.87–1.28	0.58
Education			
Low (elementary or below)	Ref		
Moderate (junior/senior high)	0.75	0.59–0.95	0.02
High (college or above)	0.79	0.56–1.11	0.18
Household income			
<US$952/month	Ref		
US$952–2222/month	1.12	0.93–1.35	0.22
>US$2222/month	1.16	0.88–1.52	0.30
Smoking status			
Never	Ref		
Current	1.24	0.96–1.60	0.10
Alcohol consumption			
No	Ref		
Less than once a week	0.71	0.53–0.94	0.02
More than once a week	0.82	0.62–1.10	0.18
Vegetable			
< 5 days/week	Ref		
5–7 days/week	0.59	0.46–0.76	< 0.001
Fruit			
< 5 days/week	Ref		
5–7 days/week	0.69	0.57–0.82	< 0.001
Betel nut			
Never	Ref		
Current	0.94	0.58–1.52	0.79
Chronic renal failure			
No	Ref		
Yes	1.46	1.03–2.08	0.04
Heart failure			
No	Ref		
Yes	1.59	1.26–2.01	< 0.001
Liver cirrhosis			
No	Ref		
Yes	1.50	0.88–2.58	0.14
Stroke			
No	Ref		
Yes	1.72	1.44–2.05	< 0.001
Cancer			
No	Ref		
Yes	1.45	1.14–1.86	0.003

^a^Dose-response relationship between numbers of tooth loss and nasogastric feeding was evaluated using the trend test (P for trend = 0.01)

*Abbreviations*: *CI* confidence interval

Sensitivity analysis was performed by excluding people with heart failure, stroke, and cancer to declare the association particularly in the healthier population. The result showed that, among older people without the three comorbidities, those with 10–28 teeth lost also had significantly increased risks of incident nasogastric feeding dependency (AHR, 1.59; 95% CI, 1.16–2.27; p-value = 0.004).

## Discussion

Our study identified a significantly increased risk of nasogastric tube feeding dependency among older adults who had lost 10–28 teeth. Moreover, older age, male gender, consuming vegetables and fruits less than 5–7 days/week, heart failure, stroke, and cancer were also significant risk factors of nasogastric feeding dependency among older adults.

Teeth loss reduces masticatory function and affects food choice, which serves as an intermittent pathway between malnutrition and diet-related chronic disease [13]. Ioannidou et al. found teeth loss predicted low protein and caloric intake as well as serum albumin level, a biomarker of malnutrition in chronic kidney disease patients [14]. Thus, nasogastric tube feeding may be a solution for nutrition supplements in patients with malnutrition. However, in a study of 891 community-dwelling Japanese elderly, Hiratsuka used serum albumin and high-sensitivity C-reactive protein levels as markers of nutritional status and systemic inflammation to evaluate the relationship between teeth loss and mortality. They found that edentulous and those individuals with 1–9 teeth had higher mortality than those with > 20 teeth. Nutritional status contributed to the association between teeth loss and mortality in individuals with teeth loss. This study may explain the parts of the biological mechanism between teeth loss and all-cause mortality in older adults [15]. Gaewkhiew reviewed 2 studies and disclosed that the number of teeth lost was associated with a smaller reduction in vegetable and fruit intake [16]. Reduction of dietary fibers increase the incidence of cardiovascular disease. The antioxidants and soluble fibers from vegetables and fruit may protect vessels from atherosclerosis [13]. The same finding was reported by Cheng et al., from a meta-analysis of prospective cohort studies, exhibiting a significant association between teeth loss and risk of cardiovascular disease and stroke [17]. Previous studies also demonstrated that teeth loss is associated with development of cognitive impairment among older adults [18]. Teeth loss also increased the risk of functional disability in the elderly population [19]. Malnutrition, cognitive impairment, and functional disability may lead to nasogastric feeding dependency.

Poor oral health results in teeth loss, which is a complex outcome that reflects of individual’s history of dental diseases and national dental service policies [20]. Poor oral health can cause systemic inflammation and immune response and thus lead to cardiovascular diseases and stroke [21]. Poor oral hygiene leads to teeth loss, which contributes to an individual’s decline in functional capacity, cognitive function, quality of life, and mortality [22–25]. A higher missing teeth count is associated with higher mortality [15, 26]. Teeth loss reduces mastication force and chewing ability, resulting in choosing soft diet, reduced nutritional intake, and poor nutrition status [13, 16]. Malnutrition may lead to sarcopenia, frailty, and increase mortality but it is reversible when effective intervention is applied.

In the past, few studies investigated the association between teeth loss and nasogastric tube feeding dependency. Previous research has shown that poor oral health (for example, inability to chew or swallow food, missing teeth, or gum disease) can impair nutrient intake (for example, eating less or low-nutrient meals), leading to poor nutrition and increasing the risk of malnutrition [27]. Malnutrition is a proven indication of nasogastric tube feeding dependency. Previous studies have also indicated that older age and comorbidities, such as dementia and stroke are significant risk factors of nasogastric feeding dependency among older adults [28].

This is a cohort study with longitudinal follow-up for 12 years, which was long enough to detect the incidence of the outcome. The NHIS dataset had collected detailed information of participants, including body mass index, socioeconomic status, and health behaviors. The NHIRD had thorough data on the medical record of the study participants. Nearly all potential confounders were included in the study. There are several limitations of our study. First, some medical conditions that may directly lead to nasogastric feeding dependency, such as parkinsonism with impaired swallowing function, oral cancer, neck and esophageal cancer, may lead to the inability to swell smoothly, causing frequent choking. Second, recall bias may be found when completing the questionnaire. Third, disease comorbidities were recorded but information on disease severity was not obtained. For example, mild stroke without affecting swallowing function may not require nasogastric tube use but severe stroke may lead to swallowing dysfunction and need nasogastric feeding. We did not measure masticatory muscle strength. Severe teeth loss may impair masticatory function, decreasing masseter muscle strength but loss of a few teeth may not. The number of teeth loss were calculated except wisdom teeth and spaces restored with implants. Removable dentures are still classified as missing teeth. Removable dentures can still help chewing function and maintain the strength of the oral muscles. This may underestimate the risk of nasogastric tube feeding dependence caused by missing teeth. Besides, nasogastric tube feeding dependence was defined by the procedure code for three or more times in the outpatient or inpatient medical records consecutively in six months. However, some people may withdraw nasogastric tube feeding six months later if they recover of swallowing function, which may overestimate the risk of tube feeding dependence.

## Conclusions

In conclusion, this study revealed that the number of teeth lost is a significant risk factor of nasogastric tube feeding dependency. It is important to maintain good oral health to prevent periodontal diseases, dental caries, and further teeth loss to prevent the occurrence of nasogastric tube feeding dependency.

Oral health plays an important part in human health. Common oral illnesses include dental caries, periodontal disease, and teeth loss, which affect about 3.47 billion people in the world [29]. Among adults with disability aged 20–80 years in Taiwan, the mean number of remaining teeth was 18.1 (SD = 10.9); 44.8% of them had less than 20 remaining teeth, including 13.7% edentulous participants [30]. Early identification of the oral disease is crucial for the prevention of the occurrence of teeth loss and the following nutrition problems [31, 32], which would reduce risk of nasogastric tube feeding dependency.

## Data Availability

The data that support the findings of this study are openly available in the Health and Welfare Data Science Center of Ministry of Health and Welfare in Taiwan at https://dep.mohw.gov.tw/dos/np-2497-113.html.
